# “Being the best person that they can be and the best mum”: a qualitative study of community volunteer doula support for disadvantaged mothers before and after birth in England

**DOI:** 10.1186/s12884-018-2170-x

**Published:** 2019-01-10

**Authors:** Jenny McLeish, Maggie Redshaw

**Affiliations:** Policy Research Unit in Maternal Health and Care, National Perinatal Epidemiology Unit, University of Oxford, Old Road Campus, Headington, Oxford, OX3 7LF UK

**Keywords:** Doula, Volunteer, Disadvantaged women, Pregnancy, Antenatal, Postnatal, Community

## Abstract

**Background:**

Disadvantaged pregnant women and new mothers are at increased risk of psychosocial stress, anxiety and depression. As well as affecting birth outcomes and child development, poor maternal emotional wellbeing can inhibit the development of parenting self-efficacy and successful adjustment to the maternal role. Social support is a protective factor against antenatal and postnatal depression, anxiety and stress, and improves mothers’ confidence in infant care. Community doula programmes have been developed to meet the social support and information needs of disadvantaged women. In these programmes trained volunteer doulas support mothers during pregnancy, at birth and for a short period postnatally.

**Methods:**

This was a descriptive qualitative study, informed by phenomenological social psychology, exploring mothers’ and doulas’ experiences of antenatal and postnatal community doula support. Semi-structured qualitative interviews were undertaken with 13 disadvantaged mothers and 19 doulas at three community volunteer doula projects in England. Interviews were audio-recorded and transcripts were analysed using inductive thematic analysis.

**Results:**

The overarching theme emerging from the analysis was “Supporting the mother to succeed and flourish”. There were five subthemes: “Overcoming stress, anxiety and unhappiness”, “Becoming knowledgeable and skilful”, “Developing self-esteem and self-efficacy”, “Using services effectively”, and “Becoming locally connected”. Doulas believed that their community role was at least as important as their role at births. Their support was highly valued by vulnerable mothers and helped to improve their parenting confidence and skills.

**Conclusions:**

Volunteer doula support before and after birth can have a positive impact on maternal emotional wellbeing, by reducing anxiety, unhappiness and stress, and increasing self-esteem and self-efficacy. Doulas help mothers feel more knowledgeable and skilful, support them to make effective use of maternity services, and enable them to build social ties in their community. To facilitate the best service for vulnerable mothers at the end of doula support, doula projects should consider formalising their relationship with other community organisations that can offer ongoing one-to-one or group support. They might also alleviate some of the potential distress caused by the ending of the doula relationship by increasing the flexibility of the ending, or by organising or permitting informal low level contact.

**Electronic supplementary material:**

The online version of this article (10.1186/s12884-018-2170-x) contains supplementary material, which is available to authorized users.

## Background

Having a baby is a major life transition involving development of a maternal identity and adjustment to the maternal role [1, 2]. An important factor in the success of this transition is the development of parenting self-efficacy, including confidence in baby care [3–5]. This can also be a time of considerable stress and poor emotional wellbeing. Pregnant and postnatal women are at increased risk of anxiety and depression if they are socially isolated, have low perceived social support, have low self-esteem, are single parents or have a poor relationship with their partner, are poor, or are under 18 [6–9]. High anxiety and depression are associated with poor birth outcomes [10]; an increased risk of adverse psychological, mental, emotional and behavioural child outcomes [11–14]; and a lower sense of efficacy as a parent [15]. Emotional distress can also inhibit successful adjustment to the maternal role [16].The highest risks of poor outcomes due to poor maternal mental health are in socio-economically disadvantaged families [17].

Psychosocial stress has been theorised as a combination of an objective stressor, a person’s subjective cognitive appraisal of the stressor, and their reaction in the light of this appraisal [18]. Pregnant women may attempt to cope with stress using emotional strategies, including positive re-appraisal, and seeking comfort or understanding; and problem-solving strategies, such as seeking information, and preparing for changes [19]. Women who feel unable to cope effectively during this life transition may feel distressed, incompetent and demoralised [20]. Stressful life events during pregnancy, and chronic stressors such as poverty, overcrowding and racism, are associated with poorer birth outcomes [10]. Pregnancy stress also increases the risk of adverse neurodevelopmental outcomes in the child [21].

Social support, that is a person’s perception of the availability of others to provide emotional, psychological and material resources [22, 23], has consistently been found to make a key contribution to the emotional wellbeing of pregnant women and new mothers. It can be a protective factor against antenatal and postnatal depression and anxiety [6–9]. It can also be a “buffer” against stress by altering appraisal of stressors, changing coping patterns and improving self-esteem and self-efficacy [23], and an ingredient in first time mothers’ confidence in infant care [24]. Community doula programmes have been developed in the United States to meet the social support needs of disadvantaged women, in particular young, first time mothers mainly from African American and Hispanic communities [25–29]. In these programmes the mother is matched to a community doula, an experienced woman who supports her during pregnancy, at birth and for a short period postnatally. The community doula’s antenatal and postnatal role is similar to that of other home visitors, but with a focus on becoming a mother [30]. In England, the first volunteer community doula project began in 2005, and by 2011 there were five volunteer doula projects working in disadvantaged communities (not exclusively with young or first time mothers) [31].

This is the first in-depth qualitative study on community doulas in England. This paper explores how the antenatal and postnatal role of the community doula is experienced and understood by the volunteer doulas and the disadvantaged women who they support. A separate paper has explored the role of these community doulas at birth [32].

## Methods

The study design, setting, recruitment, data collection methods and to great extent the analytic methods were the same as those used in looking specifically at the role of the volunteer community doula in relation to labour and birth [32]. However, the focus for this analysis is on antenatal and postnatal support.

### Study design

This qualitative descriptive study [33], was based on semi-structured, in-depth interviews, theoretically informed by phenomenological social psychology [34]. This “low-inference” [33] design was chosen because the purpose of the study was to explore participants’ own perceptions and thus to stay close to their accounts [34], while acknowledging the role of both participants’ understandings and the researchers’ interpretations in the production of knowledge [35]. The Oxford University Medical Sciences Research Ethics Committee (reference MSD-IDREC-C1–2013-111) approved the study.

### Setting

Three volunteer doula projects operating broadly the same model of doula support participated in the study. All three were based in England, in Bradford, Hull and Essex, and were chosen as areas that represented both ethnic and geographical diversity. A third sector organisation ran a project at each site which offered free doula support from women by unpaid volunteers during pregnancy, at birth, and for 6–12 weeks postnatally.

The volunteer doulas were women from the local community who had received at least 75 h initial training, leading to an accredited qualification. Regular ongoing support and supervision was provided by the project co-ordinator. The supported mothers were typically women with no partner, women whose partner was unable to be present at birth, women with additional vulnerabilities (such as social isolation, poor mental health, domestic violence, or recent migration), or women who were involved with children’s social care services. When a mother was referred by health or social care professional (or self-referred), the co-ordinator visited her to gain an understanding of her needs, and matched her with an available volunteer. Volunteer one-to-one support was provided through regular visits and telephone calls, as well as attending the birth. Contact between a volunteer doula and mother was typically weekly for approximately 1 hour, but this varied according to the mother’s needs. Support could begin at any gestational age, depending on when in pregnancy the mother was referred. If the doula was unable to attend the birth, a back-up doula would attend [32].

### Study recruitment

The co-ordinator of each volunteer doula project was contacted to introduce the research. Using the study information leaflets the co-ordinator then explained the research to the volunteers and recently supported mothers [32]. When a volunteer or mother had agreed to participate, the co-ordinator asked her permission for the researcher to contact her, or arranged an interview time. The researcher had no prior contact with participants. The sampling was purposive insofar as all participants had experience of giving or receiving volunteer doula support.

### Data collection

Semi-structured qualitative interviews were conducted between June 2015 and March 2016. Each participant was interviewed once at a time and place of the participant’s choice, after explaining the reasons for the study and obtaining written informed consent. Most chose the project base or their home, but four volunteers and one mother chose to be interviewed by telephone (oral informed consent was given and recorded in writing). Three mothers chose to have their partners present during part or all of their interviews. Although professional interpreting was offered, none of the mothers took this up; however, one mother used informal interpreting support from the project co-ordinator, and a second mother had partial informal interpreting support from her partner.

Volunteers’ interviews lasted 37–99 min (median length 54 min). Topics included their motivation for volunteering, training, activities as a doula, support received from the project, experience of working alongside health and social care professionals, and impacts on supported women and on the volunteer (see Additional file 1). Mothers’ interviews lasted 25–75 min (median length 40 min). Topics included their experience of using the maternity services, the support they had received from the doula, the impact of the doula support, and their opinion of the doula support (see Additional file 2). All interviews were audio-recorded and fully professionally transcribed, with each participant being given an anonymous identifier beginning with ‘D’ (for doulas) or ‘M’ (for mothers). Data collection continued until saturation was reached in the themes identified in the analysis.

### Data analysis

The volunteers’ and mothers’ transcripts were analysed as separate data sets using inductive thematic analysis [36]. After checking against the audio recording, each transcript was read and reread, and codes were identified inductively and recorded using NVIVO software. Codes were refined, combined and disaggregated as data collection continued, and emergent themes identified; the technique of constant comparison [37] was used to reconsider earlier codes and emergent themes in the light of subsequent interviews. Themes emerging from each data set that related to support during pregnancy and in the postnatal period were compared with the themes identified from the other data set and integrated into an overall thematic analysis. To ensure the validity of the analysis, each researcher analysed the transcripts independently; codes and emerging themes were discussed and agreed. Both researchers approached the analysis reflexively, putting aside their existing knowledge as experienced researchers in this field so that the analysis remained close to participants’ accounts, and acknowledging the potential impact of their own perspectives as White, UK-born women with children.

### Participants

Sixteen mothers and nineteen doulas took part in the study. Three of these mothers were excluded from this analysis of experiences in the antenatal and postnatal periods because they were experienced mothers without additional vulnerabilities who only needed doula support at birth. The remaining 13 mothers were from diverse backgrounds. Six had a husband or partner and seven were single parents. They ranged in age from 20 years old to mid-40’s, with the majority being in their 30’s. Three were first time mothers and 10 had 1–5 older children (mode = 1). Four were White British, one was British Asian, and eight were born abroad in Asia, Africa, and the Middle East. All had additional vulnerabilities including social isolation, poverty, poor mental health, domestic violence, recent migration (including two resettled refugees), previous traumatic birth, and an older child with disabilities.

All the doulas had experience of supporting disadvantaged mothers during pregnancy and postnatally. They had volunteered for between 4 months and 6 years (mean 3.2 years) and had each supported 2–25 families (mean 10). Fourteen were White British and five were Asian or British Asian. They ranged in age from early twenties to mid-60’s, with the majority being in their 30’s. All but one had children of their own, and three were grandmothers. Besides volunteering, 14 were in paid work, three were full time mothers, one was a student, and one was retired.

## Results

The overarching theme emerging from this analysis of the data about the community doula role working with disadvantaged mothers during pregnancy and after birth was “Supporting the mother to succeed and flourish”, described by one doula as *“being the best person that they can be and the best mum at the same time”* (D08). Doulas believed that their community role was at least as important as their role at births and that they were providing *“a short term intervention with long term impact”* (D12).

There were five subthemes: “Overcoming stress, anxiety and unhappiness”, “Becoming knowledgeable and skilful”, “Developing self-esteem and self-efficacy”, “Using services effectively”, and “Becoming locally connected”. These subthemes and the doula activities contributing them are shown in Table 1.

**Table 1:** Issues, doula activities, identified overarching theme and subthemes

Issues for pregnant women and new mothers	*Volunteer doula activities*	Subthemes, reflecting potential impact on women	Overarching theme
*Low mood* *Anxiety* *Stress*	Non-judgmental, non-professional active listening Relationship-building Emotional availability	***Overcoming stress, anxiety and unhappiness***	Supporting the mother to succeed and flourish
*Lack of information* *No family from whom to learn skills*	Providing evidence based information on pregnancy, birth, baby care Supporting development of parenting skills Not giving advice Giving relevant public health messages	***Becoming knowledgeable and skilful***	
*Poor self-esteem* *Lack of confidence* *Low self-efficacy* *Undervalued and overwhelmed*	Giving time, attention, respect Affirmation Respecting choices Showing care and concern Supporting manageable steps to success	***Developing self-esteem and self-efficacy***	
*Unfamiliarity with healthcare system* *Lack of confidence* *Difficulties communicating with healthcare professionals*	Accompanying Helping with questions Helping with understanding	***Using services effectively***	
*Social isolation* *Low awareness of services*	Facilitating links with the community Supporting social contact with other mothers	***Becoming locally connected***	

### Overcoming stress, anxiety and unhappiness

All the mothers described feeling extremely anxious during pregnancy about the impending birth, in particular the prospect of birthing without a partner present. Getting to know a doula who would support them allayed these fears: “*It was a huge weight lifted… I used to have sleepless nights thinking about what’s going to happen”* (M07).

Although a need for birth support was usually the primary reason for referral to the doula project, the mothers also described how the antenatal support addressed their wider feelings of stress and unhappiness (arising from extreme isolation and other social problems, or previous traumatic experiences). Both mothers and doulas repeatedly used the phrases *“just having somebody to talk to”* or *“someone that would listen to you”* to explain the doulas’ positive impact on psychological wellbeing. Doulas recognised that midwives were too busy, and mothers too diffident, to have these conversations about their troubles: *“You feel like you’re not important enough to get that extra 10 minutes where you want to discuss something*” (D16). By contrast the doulas made themselves readily available: “*She’s like a little light at the end of the phone, if anything I was worried about, I would text her”* (M06). Moreover, mothers felt able to confide in their doulas precisely because they were non-judgmental community volunteers: “*It’s not someone there to judge you… Because they’re not professional people, they’re not midwives, they’re not social services, they’re not doctors, you feel like you can talk about anything and you know nothing’s going to go anywhere”* (M09). Mothers also contrasted their families’ rejection of negative feelings with the doulas’ acceptance:

*“I could say what I wanted and not have [the doula] come back with anything…I quite often said, ‘I don’t want to be pregnant anymore, I’ve had enough now.’ And my mum’d be like…‘Don’t say things like that, it’s horrible things to say.’ … Whereas when I said that to [the doula], she was like, ‘Yeah, I understand’”.* (M11).

Postnatally, the doulas’ support shifted slightly to combine listening with nurturing the new mother: *“She just tried to make sure I was still sane after everything and just help with the baby, cuddle the baby, put her to sleep ... Just something to make my life a bit easier afterwards”* (M08). This practical support was greatly appreciated by mothers who were otherwise entirely on their own with their baby: *“She could sit with littl’un while you go and have a bath, and you know if he’d been up all night it was just like ten minutes where she could watch him or feed him”* (M01). Two mothers credited their doulas with helping them avoid the severe postnatal depression they had previously experienced: *“I felt like the fact that I didn’t get it again was down to the support I got”* (M04).

Both mothers and doulas emphasised that this emotional support came about through the formation of a trusting relationship, which many mothers characterised in very intimate terms: *“She was really like my sister, my friend, my mother”* (M16). Some mothers said it was difficult to accept support at first, because this threatened their sense of self-sufficiency: *“I don’t always let a lot of people in”* (M09). However, they all quickly began to enjoy the relationship: *“after getting to know her and her being such a lovely person”* (M07). The doulas showed some ambivalence about the nature of the relationship, with several finding it hard to define: “*a friend and not a friend”* (D16); *“basically a female friend…we’re not allowed to be friends”* (D04). Others were clear that they were *“befrienders*” (D18), or *“professional friends”* (D08), although in some cases they had nonetheless formed strong personal bonds with mothers: *“You can’t help chemistry and feelings”* (D04).

Irrespective of how they named the relationship, the doulas saw emotional closeness as instrumental in the success of their support: *“You’ve got to connect with them…If they’re not comfortable enough to open up with you then there’s not really any point you being there”* (D08). Many of the doulas had supported young mothers who were involved with social services because of child welfare concerns, and who were initially wary because they were distrustful of all services. Although sometimes the doula “*couldn’t find that key to unlock the door”* (D04), they also gave examples of success through patient persistence: *“[They’re] very distant and very hard to engage, but by the end of it…we’re very relaxed in each other’s company”* (D14).

### Becoming knowledgeable and skilful

A central aspect of the doula role in the community was ensuring that antenatally mothers had the information and skills they needed for pregnancy, birth, and baby care: *“Providing that information, giving the informed choices and make sure that they’re empowered to have the birth and feeding experience that they wanted”* (D03). They explained how to use the maternity services and the right to make choices about care: *“[The doula] explained how things going in hospital… how to make decisions.”* (M14). After the birth they also helped vulnerable mothers to develop parenting skills and gain *“a confidence in their own abilities”* (D14). For example, they helped mothers to become attuned to their babies’ ways of communicating: *“The bonding and attaching side of things … that opens parents’ eyes. It’s not just a whingy baby, your baby’s needing something”* (D09). They also gave timely support with feeding concerns; unlike health professionals, they had the time to sit with a mother and observe a whole feed: *“Really worried that she wasn’t getting enough milk…[the doula] just put my mind at ease”* (M06).

In providing information and support to develop parenting skills, the doulas were filling a gap for some first time mothers who were living far from their female relatives, from whom they might otherwise have learned: *“[The doula] helped me, I have nothing – mother, friends, sister – I have nothing”* (M15). Other mothers lacked information about birth and their babies because they were so overwhelmed with other crisis issues that *“the pregnancy is just one extra thing”* (D14).

The doulas were unanimous that the information they gave had to be evidence-based and from a reliable source (such as an NHS website or leaflet): “*Unless it’s written, proven and you’ve got in black and white; we can’t say, ‘Oh when mine were younger we used to do this’”* (D06). They shared these resources with the mothers so that it was clear the information was *“not coming from me”* (D11), and they referred any medical questions to the appropriate professionals: *“Whatever we say, we always say, ‘You need to speak to your health visitor and your midwife’”* (D04). This applied even when the doula did know the answer, for example, a doula who was also a qualified nurse explained that she would not give simple advice about taking paracetamol during pregnancy: *“I’ll say, ‘Well you have ask your midwife about the medication because I’m not sure.’ Even though if it was my friend asking me I’d be like, ‘Yeah, you’re fine with that’. That’s not my role”* (D01).

Although the doulas were careful not to give advice, this did not stop mothers from asking for it. One doula described how a mother turned to her for guidance in the presence of an obstetrician: *“She said to me, ‘What shall I do?’... I said, ‘It’s not up to me. It’s your choice, it’s your body … I’ll support you whatever you choose, but it’s got to be your choice ’”* (D06). Another acknowledged that mothers might find the doulas’ position on this frustrating: *“I’ve spoken to some mums who have said, ‘We don’t want somebody who just listens, we want someone who can guide us and tell us what we should be doing’”* (D04).

The doula training included public health messages about healthy eating, smoking, alcohol, and the benefits of breastfeeding. Their responsibility to pass on this information did not always sit easily with their woman-centred approach, and they felt it could be counter-productive to make a mother feel pressurised: “*I think if you start being pushy you’re the next one that won’t be let in the door and I think it’s better that we’re in”* (D05). However, they emphasised that they could give this information sensitively and effectively in the context of their relationship of trust with the mother:

*“The first few visits are about…building up that relationship and then once you’ve got that trust and that bond, it’s easier then to talk about the things that they maybe don’t want you to talk to them about, like stopping smoking.”* (D01)

### Developing self-esteem and self-efficacy

Doulas described how mothers often had low self-esteem and little sense of control or self-efficacy. They had worked with mothers who had a strongly negative self-perception, and mothers who worried that they would not be able to love their babies or care for them: *“I’ve had mums saying, ‘I think I’m a freak because I think this’”* (D05). The doulas accepted that they could not change the difficult conditions of these mothers’ lives, but instead helped to *“change people’s perspectives and how they view things”* (D08). They did this giving them *“a lot of time, attention and respect”* (D09), in particular by building their confidence and focusing on their strengths: *“Making the mums and dads feel, ‘I can do this!’ Instead of, ‘Oh, how am I going to do this?’”* (D09). Mothers contrasted the non-directive doula support with their experience of some professionals who focused on parenting deficits: *“We do feel judged… [by professionals] saying, ‘Oh you should be doing this’”* (M09).

The doulas identified four strategies that they used to build up mothers’ self-esteem and self-efficacy: affirmation, showing respect for choices, demonstrating care and concern, and supporting manageable steps to success. Affirmation comprised positive feedback, praise and encouragement: *“It gaved her a lot of confidence, because I was like, ‘You are doing really brilliant…look how far you’ve come’”* (D10). One doula described the impact on a young mother of hearing herself publically praised at a meeting with social services: *“I could say she did extremely well at the birth, she breastfed, she did the skin to skin, all the things that you’re supposed to do, she was really, really good at it as well. And so I said it, and she was sort of glowing”* (D07). An isolated mother recalled how her doula had motivated her to look to the future: *“‘Life not finished, you have to build yourself…you can do everything, go forward.’ Then I start thinking what I can do”* (M16).

The doulas had a stance of absolute respect for mothers’ decisions: *“We give the information, you make your choices, we will support your choices”* (D09). They believed that this unconditional support was important for developing mothers’ self-worth and self-belief: *“They get their self-esteem back…knowing that they can do this and people do respect their choices”* (D06). This was felt to be particularly salient for the most vulnerable mothers who had experienced little autonomy: “*They can feel knowledgeable about what’s going to happen, and they’ve got the power to make choices …a lot of the women I’ve supported haven’t been in control of their life whatsoever”* (D14).

The doulas believed that their care and concern could have a profound effect on the self-esteem of mothers who had no one else to care about them and did not believe they were worth caring about: *“How needy they were, just to have another person who was bothered about them… A lot of them say to me, ‘Why are you wasting all your time here with me?’”* (D14). The fact that the doulas were volunteers giving up their own time made this especially meaningful to the mothers: *“Even [the doula’s] kids that are sacrificing that mummy time with her, it’s the family as a whole that are supporting one person to give comfort to a total stranger… It was so overwhelming”* (M07).

Doulas had various ways of enabling mothers with low-self-efficacy to take manageable steps towards *“success at the end”* (D07). Some used goal-setting to identify priorities and the actions mothers could take towards achieving their goals, so they could “*be proud of their own achievements”* (D14). Others helped mothers to accomplish tasks they found daunting (such as phone calls to the authorities, using an escalator for the first time, or shopping in a supermarket): *“Empowering them to do it themselves, not doing it for them but be there supporting them in what they’re doing”* (D06). This built mothers’ confidence by “*showing them that they* *can* *actually do what they didn’t think they could do”* (D19). A typical technique was to gradually reduce the level of assistance offered with a task, for example, accompanying a mother to use public transport: *“It started off I was taking her, so she knows where she’s going…then she took a bus and then I met her at the bus stop, and then gradually, I met her at the fitness centre”* (D09). For young mothers who were involved with social care services, the doula’s role might stretch to teaching and encouraging basic life skills such as house cleaning, to prevent escalation of child welfare concerns:

*“A lot of the places are very dirty… they haven’t been told how to clean… I said, ‘You don’t want [social workers] saying, ‘This baby’s at risk.’ You’re good parents, you just need a little bit of a push in the right direction… What do you think we can do to sort it out quickly?’”* (D07).

### Using services effectively

Doulas accompanied mothers to their antenatal and postnatal appointments. As well as the practical help in many cases of giving them a lift by car, this was partly for moral support: *“It was just like having a sister there … she tried to get me to not think about whatever it is they were doing, they were poking and prodding everywhere”* (M08). It also enabled mothers to get more out of appointments, by supporting effective communication between mothers and clinicians.

Both doulas and mothers described how, in the stress of medical appointments, important questions were easily forgotten. The doula filled in these gaps based on previous conversations with the mother, or encouraged mothers to prepare by writing things down: *“I would have some questions and if I forgot to ask any she would say, ‘What about this?’”* (M01). Alternatively mothers might need support to ask their questions if they lacked confidence: “*All the questions that I had, I didn’t used to ask”* (M07). It could be hard for mothers to understand the technical language used in maternity care, and after the appointment doulas would review what had been said and the maternity notes: *“If mum’s not sure of any slang they use, we’ll break that down into layman’s terms for them, simplify it”* (D09). Sometimes during the appointment itself they had to *“translate it into maybe simpler terms”* (D03)*.* More commonly they facilitated direct communication by checking the mother’s understanding and asking for a clearer explanation: *“If the doctor or the midwife’s not explained something enough... I’ll ask mum, ‘Do you want any more information?’ And if she feels she wants to then we probe that with the midwife”* (D11).

### Becoming locally connected

Because the postnatal period of support was limited to 6–12 weeks, doulas had to carefully plan their exit strategy: *“As soon as the baby’s born you start thinking about leaving … how do we get mum into a position where she is okay so that you can leave them?”* (D05). The goal was to be a *“non-dependency service”* (D09) so that when the doula support ended, mothers felt able to thrive without it: *“To stand on their own two feet… and say, ‘Bye, thanks for everything,’ and not ‘Oh can you please come back?’”*(D06).

To mitigate the risk that vulnerable mothers might become dependent on their visits for social contact when “*I might be the only person that that lady might see all week”* (D01), doulas encouraged mothers to become “*integrated into their community…making other friends with babies of the same age”* (D03). They did this by connecting them to local sources of social support such as groups at children’s centres: *“Before [the doula] left we’d gone out and seen what playgroups there was, or baby massage and stuff, so when she’d left I had all this to do”* (M01). One doula project ran its own social groups for new mothers.

As well as leaving mothers socially connected, the doulas aimed to ensure that mothers were in touch with any local services they might need: *“I wasn’t leaving her in the lurch, I’d got other people involved, and she had different support workers coming to see her”* (D13). They emphasised to mothers that moving on from doula support was a sign of their capability: *“The fact that I’m leaving shows that you’ve taken a step up, and look what you can do now!”* (D14). At the same time there was occasional flexibility about the endings where the mother’s needs were very complex and would not be met by other services: *“I don’t feel like I can leave them until I feel confident that they’ve got something else in place, and actually they feel like they’re ready for motherhood and ready for life”* (D14).

All of the doulas who volunteered in the site with a 12 week postnatal period felt that this was usually long enough, in particular to pick up symptoms of postnatal depression and ensure the mother was receiving appropriate treatment or support. Doulas at the sites with a 6 week period tended to have more concerns about leaving their most vulnerable mothers: *“There are some families where… you don’t feel like you’ve left them in a good place. And you worry for a long time afterwards”* (D05). Generally they were able to rationalise these concerns within the concept of the time-limited doula role: *“It’s just not healthy for them to have this person to be constantly leaning on... you’d hinder them if you continued giving that support”* (D15). However, most doulas acknowledged that they found some endings emotionally challenging: *“You feel really attached to them… it is hard to say goodbye”* (D17).

The mothers had a different perspective on the ending of support. Making friends locally and being in touch with other services did not replace the important role the doula had played in their lives, and many became upset when recollecting saying goodbye: *“I really still missing her…the person I like, I have to lose them at the end, so that’s my struggle”* (M15). Many said that although they understood the support had to end, they did not agree that this should mean there could be no further contact at all: *“I do think when you’ve shared such an intimate time with somebody like that, it’s quite harsh just to be cut off”* (M06). Several asked during the interview for a message of greeting or thanks to be passed to their doula. Some said they wished they could give their doula a photo or invite her to their child’s first birthday; typically this was framed in terms of wanting to thank the doula for her support, and to acknowledge the reality of the relationship that had formed: *“Just a few texts here and there, to show appreciation of how much they did help you and what a difference they did make to your life”* (M07). One doula project had introduced an annual meet up for all doulas and mothers together, and this was very popular as a means to fulfil this desire.

## Discussion

The doulas’ activities during the antenatal and postnatal periods contributed towards enabling vulnerable mothers, who experienced significant stress and were at risk of poor mental health, to grow in confidence and ultimately to feel able to flourish as parents. The majority of doula literature is concerned with the doula’s role in preparing mothers for, and supporting them during birth, and primarily focuses on physical outcomes and birth experiences [38]. The five themes identified in this analysis are, however, consistent with the literatures on employed community doulas for young mothers in the USA [25, 26, 28, 30], on volunteer community doulas in the UK [39, 40], and on non-doula volunteer support during the perinatal period [41–44].

Unlike some community doula models in the United States, the volunteer doulas did not have a narrowly defined client group and did not follow a structured programme [28, 45]. Instead they worked across the emotional, informational, affirmational and instrumental dimensions of social support [46], tailored to the mothers’ diverse individual needs. Emotional support was provided through active listening and giving mothers *“time, attention and respect”*. The opportunity to talk about their difficult feelings to a non-judgemental listener, who was neither a professional nor a family member, has previously been identified as key to improving mothers’ psychological wellbeing [47–50]. Being encouraged to make choices, and having those choices unconditionally accepted, had a positive impact on self-esteem [25, 51]. In addition, a mother’s self-esteem could be enhanced by the very fact that a volunteer was willing to give her own time over many weeks, demonstrating that the mother was worthy of care and concern [47].

As well as the direct impact of the relationship on emotional wellbeing, the doulas built up vulnerable mothers’ self-efficacy, “buffering” them against stress [23]. Using a strengths-based approach, the doulas consistently affirmed and praised mothers, reframed their perception of situations [18], and enabled them to accomplish challenging tasks by giving “just enough help” to succeed [52]. They had the time to help mothers develop the confidence and parenting skills they needed, through information, demonstration, modelling, and giving mothers’ feedback on their own efforts [24]. There were times when to help a mother succeed, it was necessary for the support to address areas not strictly related to having a baby (for example, basic life skills), a process characterised by Gentry et al. [25] as “going beyond the call of doula”.

The doulas were clear about the non-clinical parameters of their role: providing evidence-based information from approved sources but always referring the mother to their midwife or doctor for medical questions or advice. This is in contrast the findings of studies about the role of doulas privately hired by mothers, where health professionals criticised doulas for failing to observe this boundary [38, 53–56]. Professional standards require midwives to use language that women can understand, and to take responsibility for checking women’s understanding [57]. However, in this study the doulas described needing to support disadvantaged mothers to make effective use of their antenatal care, because they often did not understand the technical language and lacked the confidence to ask questions [25, 38]. There was potential tension between the doulas’ woman-centred, needs-led approach to information-giving, and their role as providers of public health information about issues such as smoking, alcohol and diet [58]. The doulas had generally resolved this by situating the difficult conversations within the context of an established relationship, and by maintaining their stance of respect for whatever choice a mother chose to make once she had the information.

As with other community doula and perinatal volunteer support projects, the foundation of doula support was the establishment of a relationship of trust [26, 30, 41–44]. In contrast to many of the community doula projects in the United States [25, 26, 28], the relationships were not predicated on shared ethnicity. Other studies have also found that it can be challenging (and rewarding) to make these relationships successfully with adolescent mothers [30, 31].

Both doulas and mothers found it difficult to articulate the exact nature of this relationship. A survey of mothers and doulas in the English community doula model found that three quarters of mothers saw their doula “like a friend” [39] and 79% of doulas saw friendship in some form as part of their role [40]. The doulas in this study described emotional closeness as instrumental in the success of their support and some developed real feelings of friendship, mirroring the findings of Spiby et al. [40]. As also reported by Darwin et al. [39], many mothers experienced the relationship as special and intimate and had feelings of grief about its total withdrawal even if they were feeling confident in practical ways *“to stand on their own two feet*”. The strict rules about ceasing contact at the end of support contrast with some other perinatal volunteer support projects, which allow informal social contact to continue after support has ended. This approach can be experienced as affirming the genuineness of the relationship that has formed [41].

Darwin et al. recommend considering the extension of doula support beyond the emotionally high-risk sixth week postpartum [39], and Gentry et al. recommend that a form of support should continue beyond 3 months for adolescent mothers [25]. In one U.S. model, doula support is integrated into a wider early childhood programme that offers ongoing home visiting or group-based activities [45]. Although none of the three doula projects in this study was formally linked to an ongoing childhood programme, one was based in a children’s centre that offered groups for parents, another had extended the period of support to 12 weeks and offered its own groups for parents, and all three referred mothers to other community organisations (where these existed).

Breedlove [26] argues that the extended relational caring offered by community doulas has the potential for even greater impact than their birth support, and the doulas in this study likewise believed that they were *“a short term intervention with long term impact”*. Long term impact on the mother could be achieved through improved emotional wellbeing, increased coping skills, and a sense of successful life transition [20], enabling the mother to become “*the best person that they can be and the best mum at the same time”*. Long term impact on the child could be could be achieved through the improved outcomes from positive maternal mental health [11–14], and reduced maternal stress [21]. The doulas gave specific support to help mothers with parenting tasks such as breastfeeding, which protects child and maternal health [59], and also to help them understand and respond sensitively to their baby’s cues, which can enhance relationship-building and attachment [28, 60]. More fundamentally, the doulas “mothered the mother” [61]: they modelled a relationship of unconditional positive regard [25, 62] and the helping technique of “scaffolding” (helping the other learn by providing just enough support) [52], both of which are key ingredients in positive parenting.

### Strengths and limitations

It was a strength of this study that it was based on in-depth interviews with volunteer doulas and a diverse and disadvantaged group of mothers, enabling the two perspectives to be analysed in parallel. There were also some limitations. None of the mothers involved with children’s social services were willing to be interviewed. Two mothers who would have benefited from professional interpreting did not take up the offer, preferring to use informal interpreting support. It may be that these mothers had confidence in this arrangement and preferred not to introduce a further unknown individual into the interview context. Mothers’ partners were not participants in the study. As with other qualitative studies, these findings may not be generalizable to other populations.

## Conclusion

In addition to their role as birth supporters, the work of volunteer community doulas in the antenatal and postnatal periods is highly valued by vulnerable mothers and can help to improve their parenting confidence and skills. Mothers and doulas described positive impacts on maternal emotional wellbeing, with a reduction in anxiety, unhappiness and stress, and increases in self-esteem and self-efficacy. Mothers felt more knowledgeable and skilful, were supported to make effective use of maternity services, and were enabled to build social ties in their community.

Based on this evidence, there are clearly potential benefits to setting up and running such schemes.

To facilitate the best service for vulnerable mothers at the end of doula support, doula projects should consider formalising their relationship with other community organisations that can offer ongoing one-to-one or group support. They might also alleviate some of the potential distress caused by the ending of the doula relationship by increasing the flexibility of the ending, or by organising or permitting informal low level contact.

## Additional files

Additional file 1: Topic guide v5 doula support - doulas.docx. Interview topic guide for volunteer doulas providing community support who were interviewed (DOCX 46 kb)

Additional file 2: Topic guide v5 doula support - women.docx. Interview topic guide for women interviewed, who received volunteer doula support (DOCX 46 kb)

## Abbreviations

D: Doula
M: Mother
UK: United Kingdom
USA: United States of America

## References

[1] Mercer RT (2004). Becoming a mother versus maternal role attainment. J Nurs Scholarsh.

[2] Rubin R (1967). Attainment of the maternal role: part I. processes. Nurs Res.

[3] Bandura A, McClelland DC (1977). Social learning theory. Englewood cliffs.

[4] Barclay L, Everitt L, Rogan F, Schmied V, Wyllie A (1997). Becoming a mother--an analysis of women's experience of early motherhood. J Adv Nurs.

[5] Meleis AI, Sawyer LM, Im EO, Hilfinger Messias DK, Schumacher K (2000). Experiencing transitions: an emerging middle-range theory. Adv Nurs Sci.

[6] Robertson E, Grace S, Wallington T, Stewart DE (2004). Antenatal risk factors for postpartum depression: a synthesis of recent literature. Gen Hosp Psychiatry.

[7] Leigh B, Milgrom J (2008). Risk factors for antenatal depression, postnatal depression and parenting stress. BMC Psychiatry.

[8] Lee AM, Lam SK, Sze Mun Lau SM, Chong CS, Chui HW, Fong DY (2007). Prevalence, course, and risk factors for antenatal anxiety and depression. Obstet Gynecol.

[9] Littleton HL, Breitkopf CR, Berenson AB (2007). Correlates of anxiety symptoms during pregnancy and association with perinatal outcomes: a meta-analysis. Am J Obstet Gynecol.

[10] Dunkel Schetter C, Tanner L (2012). Anxiety, depression and stress in pregnancy: implications for mothers, children, research, and practice. Curr Opin Psychiatry.

[11] Laurent HK, Leve LD, Neiderhiser JM, Natsuaki MN, Shaw DS, Harold GT, Reiss D (2013). Effects of prenatal and postnatal parent depressive symptoms on adopted child HPA regulation: independent and moderated influences. Dev Psychol.

[12] Murray L (1992). The impact of postnatal depression on infant development. J Child Psychol Psyc.

[13] Sutter-Dallay AL, Murray L, Dequae-Merchadou L, Glatigny-Dallay E, Bourgeois ML, Verdoux H (2011). A prospective longitudinal study of the impact of early postnatal vs. chronic maternal depressive symptoms on child development. Eur Psychiatry.

[14] O'Connor TG, Heron J, Golding J, Beveridge M, Glover V (2002). Maternal antenatal anxiety and children's behavioural/emotional problems at 4 years. Report from the Avon longitudinal study of parents and children. Br J Psychiatry.

[15] Porter CL, Hsu HC (2003). First-time mothers' perceptions of efficacy during the transition to motherhood: links to infant temperament. J Fam Psychol.

[16] Emmanuel EN, Creedy DK, St John W, Brown C (2011). Maternal role development: the impact of maternal distress and social support following childbirth. Midwifery.

[17] Pearson RM, Evans J, Kounali D, Lewis G, Heron J, Ramchandani PG, O’Connor TG, Stein A. Maternal depression during pregnancy and the postnatal period: risks and possible mechanisms for offspring depression at age 18 years. JAMA Psychiatry. 2013;70. 10.1001/jamapsychiatry.2013.2163 PMC3930009 24108418

[18] Lazarus RS (1993). Coping theory and research: past, present, and future. Psychosom Med.

[19] Lobel M, Hamilton JG, Cannella DT (2008). Psychosocial perspectives on pregnancy: prenatal maternal stress and coping. Soc Personal Psychol Compass.

[20] Bobevski I, Rowe H, Clarke DM, McKenzie DP, Fisher J (2015). Early postnatal demoralisation among primiparous women in the community: measurement, prevalence and associated factors. BMC Pregnancy Childbirth.

[21] O'Donnell K, O'Connor TG, Glover V (2009). Prenatal stress and neurodevelopment of the child: focus on the HPA axis and role of the placenta. Dev Neurosci.

[22] Priel B, Besser A (2002). Perceptions of early relationships during the transition to motherhood: the mediating role of social support. Inf Ment Health J.

[23] Cohen S, Wills TA (1985). Stress, social support, and the buffering hypothesis. Psychol Bull.

[24] Leahy Warren P (2005). First-time mothers: social support and confidence in infant care. J Adv Nurs.

[25] Gentry QM, Nolte KM, Gonzalez A, Pearson M, Ivey S (2010). "going beyond the call of doula": a grounded theory analysis of the diverse roles community-based doulas play in the lives of pregnant and parenting adolescent mothers. J Perinat Educ.

[26] Breedlove G (2005). Perceptions of social support from pregnant and parenting teens using community-based doulas. J Perinatal Educ.

[27] Deitrick L, Draves P (2008). Attitudes towards doula support during pregnancy by clients, doulas, and labor-and-delivery nurses: a case study from Tampa, Florida. Hum Organ.

[28] Hans SL, Thullen M, Henson LG, Lee H, Edwards RC, Bernstein VJ (2013). Promoting positive mother-infant relationships: a randomized trial of community doula support for young mothers: community doula randomized trial. Inf Ment Health J.

[29] Kane Low L, Moffat A, Brennan P (2006). Doulas as community health workers: lessons learned from a volunteer program. J Perinat Educ.

[30] Humphries ML, Korfmacher J (2012). The good, the bad, and the ambivalent: quality of alliance in a support program for young mothers. Inf Ment Health J.

[31] Spiby H, Green JM, Darwin Z, Willmot H, Knox D, McLeish J, Smith M. Multisite implementation of trained volunteer doula support for disadvantaged childbearing women: a mixed-methods evaluation. Health Services and Delivery Research. 2015;3(8). 25834865

[32] McLeish J, Redshaw M (2017). A qualitative study of volunteer doulas working alongside midwives at births in England: Mothers' and doulas' experiences. Midwifery.

[33] Sandelowski M (2000). Whatever happened to qualitative description?. Res Nurs Health.

[34] Landridge D (2008). Phenomenology and critical social psychology: directions and debates in theory and research. Soc Personal Psychol Compass.

[35] Pidgeon N, Henwood K: Using grounded theory in psychological research. In: *Doing qualitative analysis in psychology.* Edited by Hayes N. Hove: Psychology Press; 1997.

[36] Braun V, Clarke V (2006). Using thematic analysis in psychology. Qual Res Psych.

[37] Glaser B, Strauss A (1967). The discovery of grounded theory. Strategies for qualitative research.

[38] Steel A, Frawley J, Adams J, Diezel H (2015). Trained or professional doulas in the support and care of pregnant and birthing women: a critical integrative review. Health Soc Care Community.

[39] Darwin Z, Green J, McLeish J, Willmot H, Spiby H (2016). Evaluation of trained volunteer doula services for disadvantaged women in five areas in England: women's experiences. Health Soc Care Community.

[40] Spiby H, McLeish J, Green J, Darwin Z (2016). The greatest feeling you get, knowing you have made a big difference': survey findings on the motivation and experiences of trained volunteer doulas in England. BMC Pregnancy Childbirth.

[41] McLeish J, Redshaw M (2015). Peer support during pregnancy and early parenthood: a qualitative study of models and perceptions. BMC Pregnancy Childbirth.

[42] Suppiah C (2008). A collective evaluation of community parent Programmes: what works well and in what circumstances? The Health Foundation, NHS south West Essex, parents 1st.

[43] Small R, Taft AJ, Brown SJ (2011). The power of social connection and support in improving health: lessons from social support interventions with childbearing women. BMC Public Health.

[44] Barlow J, Coe C (2012). Family action perinatal support project, research findings report.

[45] Ounce of Prevention Fund (2005). The first days of life: adding doulas to early childhood programs.

[46] Brown MA (1986). Social support during pregnancy: a unidimensional or multidimensional construct?. Nurs Res.

[47] McLeish J, Redshaw M (2017). Mothers’ accounts of the impact on emotional wellbeing of organised peer support in pregnancy and early parenthood: a qualitative study. BMC Pregnancy Childbirth.

[48] Taggart AV, Short SD, Barclay L (2000). She has made me feel human again': an evaluation of a volunteer home-based visiting project for mothers. Health Soc Care Community.

[49] Frost N, Johnson L, Stein M, Wallis L (2000). Home-start and the delivery of family support. Child Soc.

[50] MacPherson K, Barnes J, Nichols M, Dixon S. Volunteer support for mothers with new babies: perceptions of need and support received. Child Soc. 2009.

[51] Kozhimannil KB, Vogelsang CA, Hardeman RR, Prasad S (2016). Disrupting the pathways of social determinants of health: doula support during pregnancy and childbirth. J Am Board Fam Med.

[52] Wood D, Bruner JS, Ross G (1976). The role of tutoring in problem solving. J Child Psych Psychiatry.

[53] Ballen LE, Fulcher AJ (2006). Nurses and doulas: complementary roles to provide optimal maternity care. J Obstet Gynecol Neonatal Nurs.

[54] Campbell-Voytal K, Fry McComish J, Visger JM, Rowland CA, Kelleher J (2011). Postpartum doulas: motivations and perceptions of practice. Midwifery.

[55] Papagni K, Buckner E (2006). Doula support and attitudes of intrapartum nurses: a qualitative study from the Patient's perspective. J Perinat Educ.

[56] Stevens J, Dahlen H, Peters K, Jackson D (2011). Midwives’ and doulas’ perspectives of the role of the doula in Australia: a qualitative study. Midwifery.

[57] Nursing and Midwifery Council (2015). The code. Professional standards of practice and behaviour for nurses and midwives. London.

[58] McLeish J, Spiby H, Darwin Z, Willmot H, Green J (2016). The processes of implementing and sustaining an intensive volunteer one-to-one support (doula) service for disadvantaged pregnant women. Voluntary Sector Review.

[59] Victora CG, Bahl R, Barros AJD, França GVA, Horton S, Krasevec J, Murch S, Sankar MJ, Walker N, Rollins NC (2017). Breastfeeding in the 21st century: epidemiology, mechanisms, and lifelong effect. Lancet.

[60] Bowlby J (1969). Attachment and Loss, Vol 1: Attachment.

[61] Klaus MH, Kennell JH, Klaus PH: Mothering the mother: how a doula can help you have a shorter, easier, and healthier birth. Cambridge, MA Perseus Books; 1993.

[62] Rogers C (1956). Client-centered therapy.

