# Supplementary material for: “Being the best person that they can be and the best mum”: a qualitative study of community volunteer doula support for disadvantaged mothers before and after birth in England
Source: BMC Pregnancy Childbirth. 2019 Jan 10;19:21. doi: 10.1186/s12884-018-2170-x (PMC6327467; doi:10.1186/s12884-018-2170-x)
Supplement: Supplementary file 1 — Topic guide v5 doula support - doulas.docx. Interview topic guide for volunteer doulas providing community support who were interviewed (DOCX 46 kb) [file 12884_2018_2170_MOESM1_ESM.docx]

**
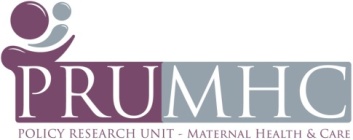

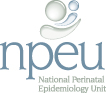
**

**Topic Guide for doula support interviews - Doulas**

1. **Background questions**
   - age, first language, family, time in UK/area, other work/study/volunteering, how long a doula
2. **The doula role**

- Can you tell me what being a doula means for you?
- How do you know if you’ve “succeeded”?/ what makes it a good experience?

1. **Deciding to become a doula**
   - How did you hear about the doula project?
   - What made you decide to become a doula?
2. **The doula training**
   - What do you think of the training to help you? (how ready were you to start volunteering at the end of training?)
   - What did you like best about the training?
   - Is there anything you would you change?
3. **Activity as a doula**
   - How many women have you supported?
     - At birth; in community; as back up?
   - Tell me about what you do as a doula – activities, time spent
     - Emotional, practical, information, confidence, public health…
   - How would you describe the relationship you have with women?
   - Tell me about the different aspects of antenatal/birth/postnatal support
   - Is there any tension between supporting a woman’s choices & your role in health promotion?
   - How do you feel about ending the relationship?
     - What do you do to prepare women for the end?
   - How does it work with or being a back up doula?
4. **Impact of doula support on women**
   - What do you think the women you support gain?
   - What do you think makes doula support work? (relationship, the training, being a volunteer…)
   - What’s it like working with a couple?
5. **Doula support and other services**
   - How do you feel doula support is different from other services?
   - How does it fit in with other services?
   - How (well) do you work with midwives or other maternity providers at birth?
6. **Positive and less positive aspects of being a doula**
   - Tell me about something you are especially proud of as a doula

- Is there anything that hasn’t gone the way you would hope?
- Has anything surprised you about being a doula?

1. **Impact of being a doula on you**
   - What have you gained from being a doula?
   - Has your being a doula affected your family? (eg being on call)
   - Has being a doula affected your plans for the future?
   - What keeps you involved?
2. **Support from the doula project**
   - How well do you feel the co-ordinator supports doulas when needed?
     - Have you ever needed emotional support?
   - Is there peer support among the doulas?
3. **Advice to others thinking of becoming a doula**
   - What advice would you give someone else thinking of becoming a doula?
4. **Best and worst things**

**To sum up…**

- - What’s the most difficult thing about being a peer supporter?
  - What’s the best thing about being a peer supporter?
