# Supplementary material for: “Being the best person that they can be and the best mum”: a qualitative study of community volunteer doula support for disadvantaged mothers before and after birth in England
Source: BMC Pregnancy Childbirth. 2019 Jan 10;19:21. doi: 10.1186/s12884-018-2170-x (PMC6327467; doi:10.1186/s12884-018-2170-x)
Supplement: Supplementary file 2 — Topic guide v5 doula support - women.docx. Interview topic guide for women interviewed, who received volunteer doula support (DOCX 46 kb) [file 12884_2018_2170_MOESM2_ESM.docx]

**
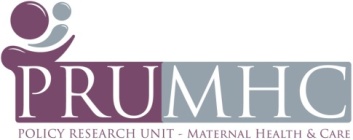

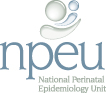
**

**Topic Guide for doula support interviews: women supported**

1. **Background questions**
   - Tell me about yourself

(age, language, family, with partner, born in UK, time in UK/in area)

1. **Having a baby/becoming a mother**
   - Tell me about having your baby/becoming a mum.
2. **Experience of maternity services**
   - What was it like for you using the maternity services?
   - Antenatal, birth, postnatal?

- What do you think about the **people** who looked after you and how they treated you?
- How did you find getting **information**/ **understanding** what was going on?
- Did you feel you had a **choice** about care? (scans, anomaly tests, pain relief, birth interventions)
- What was it like **in hospital** after the birth?
- **Religious/cultural** needs?

1. **Involvement with the doula**

- Tell me about your involvement with the doula
  - How did you come to have a doula?
    - When started/finished

1. **Doula’s role antenatal / postnatal**
   - What did the doula do with you?
     - visits, groups, signposting, birth prep, listening, information…
   - How often/for how long did you see the doula? – antenatal, birth, postnatal
   - What did the relationship feel like?
   - What was it like having a back up doula?
2. **Doula at birth**

- What was it like having the doula with you at birth?
  - Anyone else there? (if so: how did the doula work with them?)
- What did she do and how did it affect you?
- How did that work with the midwives/ doctors?

1. **Impact of doula support**
   - What difference has the doula made to you?
   - Has it helped you in the way you hoped?
2. **Voluntary nature of support**
   - Did it make a difference that the doula was a volunteer?
   - Did you see the doula as same/different from other services?
3. **Endings**
   - How did you feel about the ending of the support?
     - Timing, emotional aspects
4. **Positive and less positive aspects of having a doula**
   - What’s the best thing about the doula?
   - If there was one thing you could change about the doula support, what would that be?
   - Would you recommend the doula scheme to other mothers, and why?
